# Lentinan Attenuates Damage of the Small Intestinal Mucosa, Liver, and Lung in Mice with Gut-Origin Sepsis

**DOI:** 10.1155/2021/2052757

**Published:** 2021-11-08

**Authors:** Zhongshen Kuang, Tingting Jin, ChangYi Wu, Yanan Zong, Panpan Yin, WangYu Dong, Xue Lin, WeiYan You, Chenlong Zhang, Lijie Wang, Yongying Liu, Shan Ren, Jiangwen Yin, Hang Xu

**Affiliations:** ^1^Department of Intensive Care Unit, First Affiliated Hospital, School of Medicine, Shihezi University, Shihezi, Xinjiang 832002, China; ^2^Department of Anesthesiology, Peking University Third Hospital, Beijing 100191, China; ^3^Department of Traditional Chinese Medicine, Xiang'an Hospital of Xiamen University, Xiamen, Fujian 361100, China; ^4^Department of Anesthesiology, First Affiliated Hospital, School of Medicine, Shihezi University, Shihezi, Xinjiang 832002, China

## Abstract

This study is aimed at exploring the effects of lentinan on small intestinal mucosa as well as lung and liver injury in mice with gut-origin sepsis. Cecal ligation and perforation (CLP) were used to construct a mouse model of gut-origin sepsis. The mice were randomly divided into six groups: sham operation group (sham), gut-origin sepsis model group (CLP), ulinastatin-positive drug control group (UTI), lentinan low concentration group (LTN-L, 5 mg/kg), lentinan medium concentration group (LTN-M, 10 mg/kg), and lentinan high concentration group (LTN-H, 20 mg/kg). H&E staining was used to detect the pathological damage of the small intestine, liver, and lung. The serum of mice in each group was collected to detect the expression changes of inflammatory cytokines, oxidative stress biomarkers, and liver function indexes. In vitro assessment of bacterial translocation was achieved through inoculated culture media. Western blot and RT-qPCR were used to detect the expression of molecules related to the NF-*κ*B signaling pathway in the small intestine tissues of mice. The results showed that compared with the CLP group, the injury degree of the small intestine, liver, and lung in mice with gut-origin sepsis was improved with the increase of lentinan concentration. In addition, TNF-*α*, IL-1*β*, IL-6, and HMGB1 were decreased with the increase of lentinan concentration, but the expression of IL-10 was increased. Lentinan could also reduce the expression of oxidative stress injury indexes and liver function indexes and inhibit bacterial translocation to liver and lung tissues. Further mechanism investigation revealed that lentinan downregulated the expression of the NF-*κ*B signaling pathway molecules (NF-*κ*B, TLR4, and Bax) and upregulated the expression of occludin and Bcl-2. In conclusion, lentinan inhibits the activity of the NF-*κ*B signaling pathway, thus attenuating injuries of small intestinal mucosa and liver and lung in mice with gut-origin sepsis and reducing the inflammatory response in the process of sepsis.

## 1. Introduction

Sepsis, as one of the common critical illnesses, is a systemic inflammatory response syndrome caused by intestinal infection and associated with high mortality [1–3]. Sepsis induces dysfunction and injuries of multiple functional organs due to a dysregulated host response to infection and can be life-threatening in severe cases [4]. However, the specific mechanism of sepsis leading to organ dysfunction and injury is highly complex. Studies have shown that gut-origin sepsis caused by impaired intestinal barrier function can result in septic shock and multiple organ dysfunctions, accompanied by severe intestinal inflammatory injury [5]. And the persistent inflammation leads to a significant upregulation of intracellular reactive oxygen species and promotes the oxidative stress of organs, thus further aggravating organ injuries of patients [6]. Therefore, exploring the regulatory mechanisms of the inflammatory response and oxidative stress in gut-origin sepsis can provide a more favorable theoretical basis for clinical treatment.

Lentinan, a *β*-1,3-glucan with high medicinal value, is extracted from the mycelia of *Lentinula edodes.* It has pharmacological activities and safety such as immune regulation, antioxidation, anti-inflammatory, antimicrobial, and metabolic regulation [7]. In addition, lentinan is a biological response modifier that has the potential to protect the host from oxidative damage without significant adverse reactions [8]. In recent years, lentinan has been widely used in the adjuvant treatment of gastric cancer, lung cancer, colorectal cancer, bladder cancer, and other tumors [9, 10]. Besides, lentinan is effective in treating intestinal cancers through immunoregulation [11, 12]. Lentinan can alleviate intestinal inflammatory injury by reducing inflammatory cytokine secretion and inflammatory cell infiltration [13]. Liu et al. [14] also confirmed that lentinan could treat inflammatory bowel disease by inhibiting the TLR4 signaling pathway and restoring the intestinal microbial structure. Moreover, lentinan improves the antioxidant capacity of animals and maintains the stability of intestinal barrier function [15, 16]. Lentinan, we speculate, can treat gut-origin sepsis based on its antioxidant and anti-inflammatory effects on the intestine. Ulinastatin is a serine protease inhibitor with anti-inflammatory properties, which has been applied to treat septic liver injury and inflammation [17]. In this study, with ulinastatin as a positive control drug, we investigated the effects of lentinan on the injury of the small intestine, liver, and lung and inflammatory response in mice with gut-origin sepsis.

## 2. Methods and Materials

### 2.1. Ethics Statement

All animal experimental processes followed the *Guide for the Care and Use of Laboratory Animals* by the National Institutes of Health. The experiments were approved by the Animals Ethics Committee of the First Affiliated Hospital of Medical College, Shihezi University (2019-23).

### 2.2. Animal Experiments

Sixty C57 male mice aged 6-8 weeks and weighing 18-22 g were randomly divided into 6 groups (10 mice/group), including the sham operation group (sham), gut-origin sepsis model group (CLP), ulinastatin-positive drug control group (UTI), lentinan low concentration group (LTN-L, 5 mg/kg), lentinan medium concentration group (LTN-M, 10 mg/kg), and lentinan high concentration group (LTN-H, 20 mg/kg).

Mice were fasted for 24 h prior to model construction and then were anesthetized with sevoflurane (Solarbio, China). Subsequently, the anesthetized mice were subjected to laparotomy to expose their cecum, followed by ligation at 1/2 of the cecum. The cecum was punctured once with a 21-gauge needle at the area with sparse blood vessels, and then, a small amount of content was squeezed out, and finally, the wound was sutured. After the operation, normal saline (30 mL/kg) was injected into the mice. At 6 h after operation, the tissues and blood samples were collected. By contrast, the sham group was only given laparotomy and cecum exploration, and normal saline (30 mL/kg) was injected subcutaneously after the operation. In order to determine the effectiveness of lentinan in the treatment of gut-origin sepsis, ulinastatin was a positive control drug. At 6 h after modeling, the mice in the UTI group were injected intraperitoneally with ulinastatin (3000 U/100 g) for 1 week, once a day, and the LTN groups were injected intraperitoneally with lentinan for 1 week, once a day. The injection doses of lentinan in the LTN-L, LTN-M, and LTN-H groups were 5 mg/kg, 10 mg/kg, and 20 mg/kg, respectively. One week later, the mice were euthanized. Then, 1 cm of the distal ileum and the intestine, lung, and liver tissues of mice were collected. The peripheral blood was also extracted and stored at -80°C.

### 2.3. H&E Staining

The ileum, lung, and liver of mice were paraffin-embedded using a biological tissue embedding center (Flyde, China) and sectioned using a paraffin microtome. Subsequently, xylene (Damao, China) and absolute alcohol (Damao, China) were used for deparaffinization. Hematoxylin (Solarbio, China) and eosin staining solutions (Solarbio, China) were successively used for nuclear and cytoplasm staining. The sections were dehydrated in absolute alcohol and xylene, respectively. Finally, the sections were laid on the glass slides and mounted with neutral gum.

### 2.4. Enzyme-Linked Immunosorbent Assay (ELISA)

Serum of mice in each group was collected, and the expression of serum inflammatory factors TNF-*α*, IL-1*β*, IL-6, and HMGB1 and anti-inflammatory factor IL-10 was detected according to the instruction of ELISA Kit (Mlbio, China). The liver tissues of mice were collected and homogenized and then analyzed by enzyme-linked immune analyzer (BioTek, USA) in accordance with the instructions of Total Superoxide Dismutase Assay Kit (Beyotime, China), ELISA Kit for Myeloperoxidase for Mouse (BioVision, USA), and Lipid Peroxidation MDA Assay Kit (Beyotime, China). Each sample was analyzed in triplicate.

### 2.5. Detection of Liver Function Indexes

Serum of mice in each group was collected, and the contents of alanine transaminase (ALT), aspartate transaminase (AST), and total bilirubin (TBIL) were detected by Hitachi 7600-020 Automatic Analyzer (Hitachi High-Technologies Corporation, Tokyo, Japan). Each sample was analyzed in triplicate.

### 2.6. Analysis of Bacterial Translocation

Bacterial translocation analysis was performed as described previously [18]. Mesenteric lymph node tissues, lung tissues, and liver tissues of mice in each group were taken under aseptic conditions. After weighing, 1 mL of saline was used for homogenization, and 0.1 mL of tissue suspension was inoculated into blood agar medium, respectively, and incubated at 37°C for 24-48 h. The number of colonies was read using the plate viable bacteria counting method. The bacterial translocation rate (%) was expressed as number of colony-forming mice/number of mice per group.

### 2.7. Immunohistochemical Analysis (IHC)

The small intestine tissue sections were incubated with 0.5% troxone for 10 min and then sealed with goat serum at ambient temperature for 10 min. Subsequently, sections were incubated with NF-*κ*B antibody (ZenBio, China) and caspase-3 antibody overnight at 4°C. The antibodies were removed, and sections were rinsed 3 times with PBS (Gibco). After that, the sections were incubated with the FITC-labeled secondary antibody (CST, USA) at ambient temperature in the dark and finally covered with 50% glycerol on the glass slides. A fluorescence inverted microscope (Leica, Germany) was used to photograph. The intensity of positive staining in tissue sections was analyzed by integrated optical density (IOD) using the ImageJ 1.8.0 version (NIH, Bethesda, Maryland) as described previously [19].

### 2.8. Western Blot

Tissue proteins were extracted after homogenizing the small intestinal tissues. The denatured proteins were separated using 12% SDS-PAGE (MultiSciences Biotech, China). Subsequently, the proteins on the gel were transferred to the PDVF membranes (Bio-Rad, USA). Following the blocking step with 5% skimmed milk powder for 1 h, the membranes were incubated with primary antibody (1 500 ZenBio, China) overnight at 4°C. The membranes were then incubated with secondary antibody Goat Anti-Mouse IgG (ZenBio, China) for 1 h for protein labeling. The protein bands were developed using a chromogenic agent (Beyotime, China) and photographed in a two-color infrared laser imaging system (LI-COR, Germany). Each sample was analyzed in triplicate.

### 2.9. Statistical Analysis

All results in this study were expressed in the form of the mean ± standard deviation (SD), and all experiments were repeated three times independently. SPSS 21.00 was used for statistical analysis. The differences between groups were analyzed by *t*-test or one-way analysis of variance (ANOVA). *p* < 0.05 indicated a statistically significant difference.

## 3. Results

### 3.1. Lentinan Alleviates the Injuries of the Intestine, Liver, and Lung in Mice with Gut-Origin Sepsis

**Figure 1:**
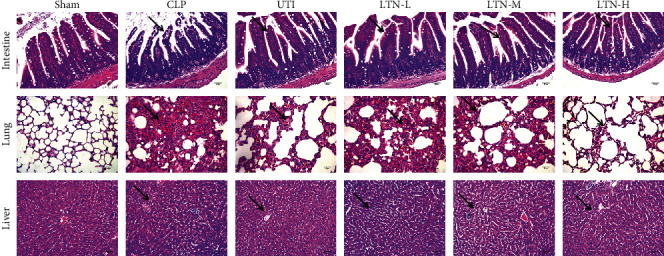
Lentinan alleviates injuries of the intestine, liver, and lung in mice with gut-origin sepsis. CLP: cecal ligation and perforation; UTI: ulinastatin; LTN-L: 5 mg/kg lentinan; LTN-M: 10 mg/kg lentinan; LTN-H: 20 mg/kg lentinan.

In order to verify the effect of lentinan on gut-origin sepsis, a mouse model of gut-origin sepsis was established. H&E staining results of small intestinal tissues (Figure 1) showed that in the CLP group, the small intestinal villi were shortened; epithelial cells and goblet cells moved toward the lumen; lamina propria was exposed; mucosal tissue was atrophied, and a large amount of inflammatory cell infiltration was observed. After ulinastatin or lentinan treatment, inflammatory cells in small intestinal tissues were reduced; villus injury in the small intestine was relieved; the epithelial cells and goblet cells were arranged more evenly.

H&E staining results of lung tissues showed that a large number of alveolar wall thickening, alveolar structural disorder, telangiectasia, alveolar hyperemia, and interstitial inflammatory cell infiltration could be seen in the CLP group. After ulinastatin or lentinan treatment, hemorrhage of lung tissue and the number of inflammatory cells were decreased, and the alveolar structure tended to be intact. Among the LTN groups, the higher the concentration of lentinan, the less the degree of lung injury.

H&E staining results of liver tissues showed that in the CLP group, obvious pathological damage could be observed, with hepatocyte swelling and neutrophil infiltration in the portal area. However, the administration of lentinan attenuated liver injury induced by CLP.

These results suggested that lentinan could improve the injuries of the small intestine, lung, and liver in mice with gut-origin sepsis, and the pathological injury was obviously alleviated with the increase of lentinan concentration.

### 3.2. Lentinan Reduces the Inflammatory Response Caused by Gut-Origin Sepsis

**Figure 2:**
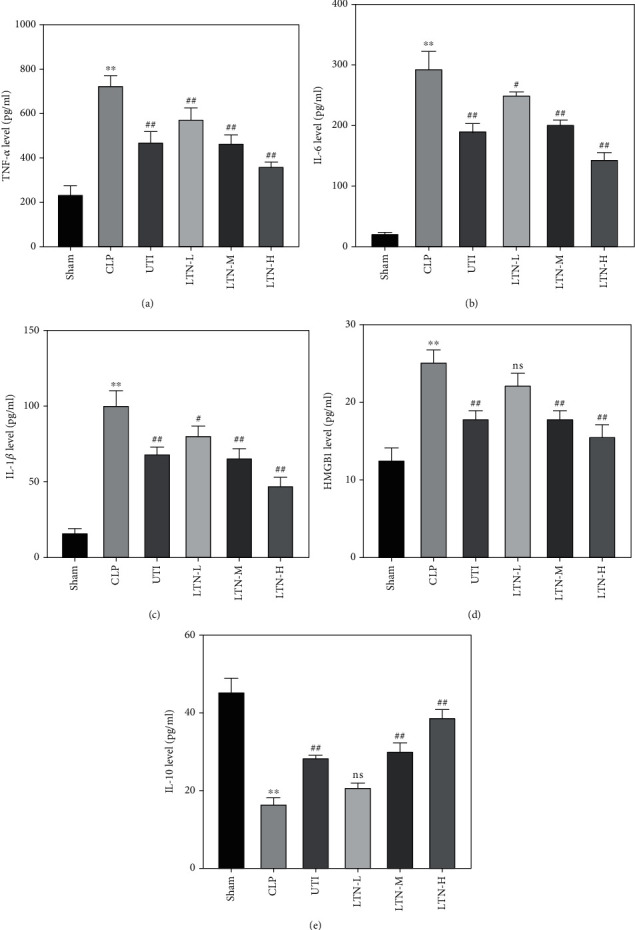
Lentinan reduces the inflammatory response caused by gut-origin sepsis. The contents of proinflammatory cytokines TNF-*α* (a), IL-6 (b), IL-1*β* (c), and HMGB1 (d) and anti-inflammatory cytokine IL-10 (e) in serum of mice were detected by ELISA. The experimental results were expressed in the form of the mean ± standard deviation (SD). ^∗∗^*p* < 0.01 vs. sham group; ^#^*p* < 0.05 and ^##^*p* < 0.01 vs. CLP group. ns: no statistical significance.

We detected the secretion of inflammatory cytokines in the serum of mice by ELISA so as to investigate the regulatory effect of lentinan on inflammatory response in mice with gut-origin sepsis. ELISA assay results showed that compared with the sham group, the expression of proinflammatory cytokines (including TNF-*α*, IL-1*β*, IL-6, and HMGB1) in serum of the CLP group was significantly increased (Figures 2(a)–2(d)), while the expression of anti-inflammatory cytokine IL-10 was significantly decreased (Figure 2(e)). After given ulinastatin or lentinan, the CLP mice showed reduced expression of TNF-*α*, IL-1*β*, IL-6, and HMGB1, but increased expression of IL-10. And with the increase of the concentration of lentinan, the expression of inflammatory factors decreased gradually while the expression of IL-10 increased. These results suggested that gut-origin sepsis aggravated the inflammatory response in mice, but lentinan could effectively reduce the inflammation.

### 3.3. Lentinan Improves Liver Injury in Mice with Gut-Origin Sepsis

**Figure 3:**
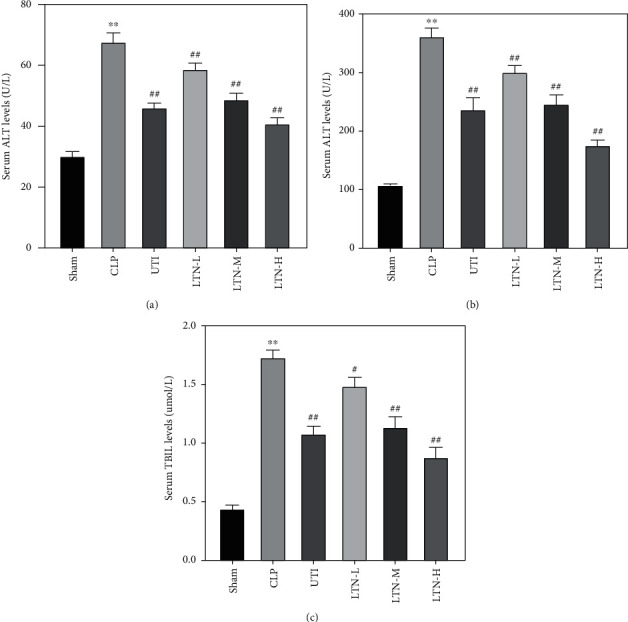
Effect of lentinan on serum ALT (a), AST (b), and TBIL (c) levels in mice with gut-origin sepsis. ALT: alanine aminotransferase; AST: aspartate aminotransferase. The experimental results were expressed in the form of the mean ± standard deviation (SD). ^∗∗^*p* < 0.01 vs. sham group. ^#^*p* < 0.05 and ^##^*p* < 0.01 vs. CLP group.

To further determine the effect of lentinan on liver injury in mice with gut-origin sepsis, the expression of ALT, AST, and TBIL in the serum was examined. As shown in Figure 3, the levels of ALT, AST, and TBIL in serum of mice in the CLP group were significantly higher than those of the sham group. Both ulinastatin or lentinan could significantly reduce the levels of ALT, AST, and TBIL in the serum of CLP mice; the effect of lentinan was dose-dependent.

### 3.4. Lentinan Reduces Peroxidase Activity in the Liver of Mice with Gut-Origin Sepsis

**Figure 4:**
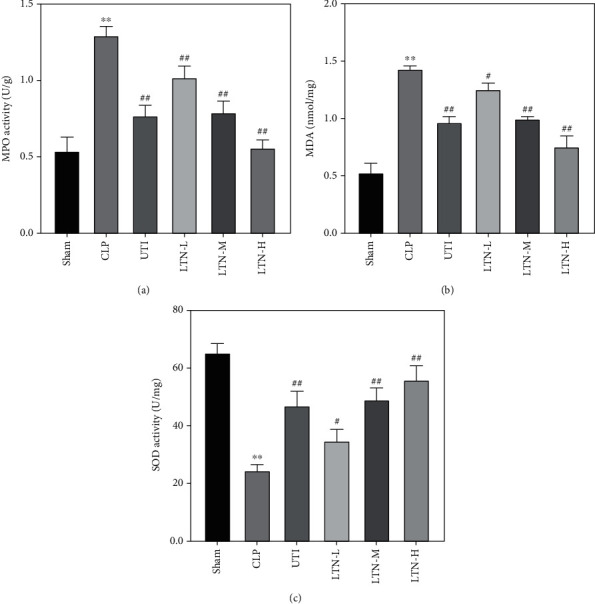
Effect of lentinan on peroxidase activity in the liver of mice with gut-origin sepsis. Detection of the contents of MPO (a), MDA (b), and SOD (c) in liver tissues of mice. The experimental results were expressed in the form of the mean ± standard deviation (SD). ^∗∗^*p* < 0.01 vs. sham group. ^#^*p* < 0.05 and ^##^*p* < 0.01 vs. CLP group.

The results of Figures 4(a)–4(c) showed that, compared with the sham group, the expression of myeloperoxidase (MPO) and malondialdehyde (MDA) in liver tissue of the CLP group was significantly upregulated, while the level of superoxide dismutase (SOD) was significantly decreased. However, after treatment with ulinastatin or lentinan, the expression of MPO and MDA was significantly reduced while the expression of SOD was increased. These results showed that gut-origin sepsis caused obvious oxidative stress injury in the liver of mice, but lentinan could effectively improve this oxidative stress injury.

### 3.5. Lentinan Inhibits Bacterial Translocation to Various Organs of Mice with Gut-Origin Sepsis

Table 1 shows that compared with the sham group, there was obvious bacterial translocation to mesenteric lymph nodes, lung, and liver tissues in the CLP group (*p* < 0.05). Both ulinastatin and lentinan treatment significantly reduced the bacterial translocation rate in various organs of mice with gut-origin sepsis, and the increase of lentinan concentration could promote this decrease.

### 3.6. Lentinan Inhibits Sepsis-Induced Activation of the NF-*κ*B Signaling Pathway

**Figure 5:**
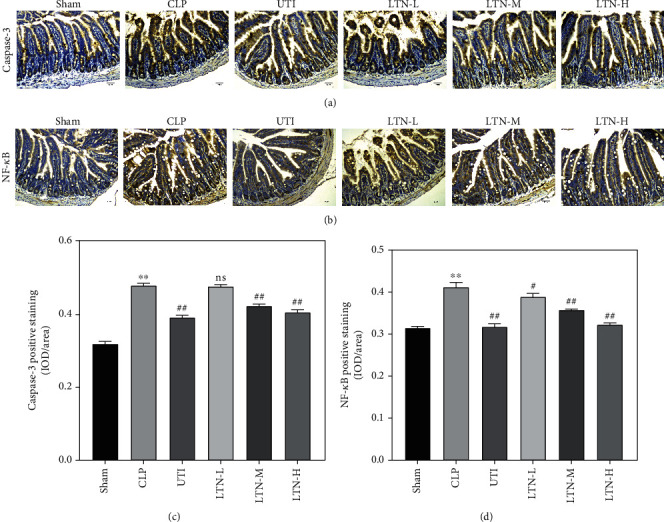
Lentinan inhibits the apoptosis of intestinal epithelial cells and decreases the expression of NF-*κ*B. (a) The expression of caspase-3 was detected by IHC in the small intestine of mice. (b) IHC assay of NF-*κ*B in the small intestine of mice. (c, d) Quantitative results of caspase-3 and NF-*κ*B. ^∗∗^*p* < 0.01 vs. sham group. ^#^*p* < 0.05 and ^##^*p* < 0.01 vs. CLP group. IOD: integrated optical density.

The specific mechanism of lentinan in improving intestinal injury in mice with gut-origin sepsis was further explored. The results (Figures 5(a) and 5(c)) showed that gut-origin sepsis induced an increase in the expression of caspase-3 in the small intestine tissue of mice, while ulinastatin and lentinan treatment could significantly inhibit expression of caspase-3 in CLP mice, and the inhibitory effect of lentinan was dose-dependent. In addition, the IHC results showed that the protein expression level of NF-*κ*B was significantly increased in the small intestine tissue of CLP mice, but its protein expression was markedly decreased after ulinastatin and lentinan treatment and the inhibitory effect of lentinan was also dose-dependent (Figures 5(b) and 5(d)). The above results indicated that lentinan might affect the apoptosis of small intestinal epithelial cells by regulating NF-*κ*B expression and thus improve intestinal damage.

**Figure 6:**
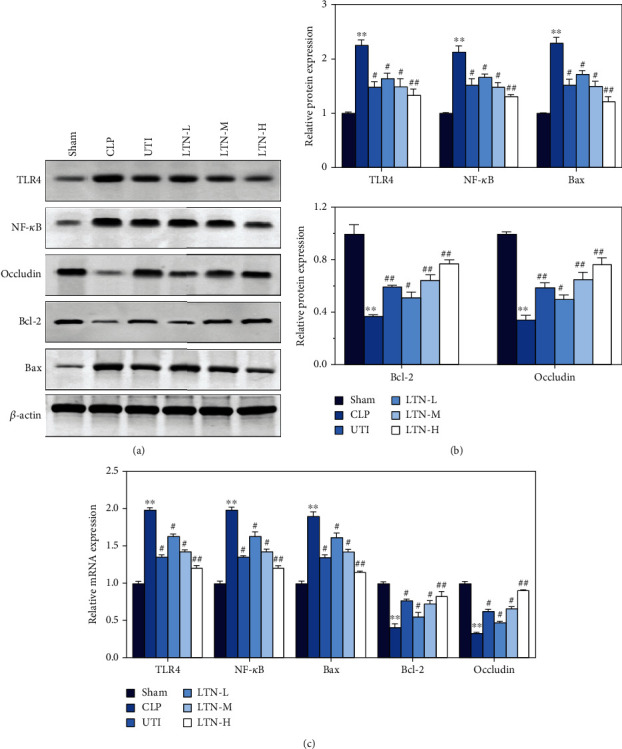
Lentinan downregulates the NF-*κ*B signaling pathway and thus inhibits apoptosis of intestinal epithelial cells. (a) Western blot was used to detect the protein expression of TLR4, NF-*κ*B, Bcl-2, occludin, and Bax in the small intestine tissue of each group. (b) Quantifications of western blots. (c) RT-qPCR was adopted for detecting the mRNA expression of TLR4, NF-*κ*B, Bcl-2, occludin, and Bax in the small intestine of each group. The experimental results were expressed in the form of the mean ± standard deviation (SD). ^∗∗^*p* < 0.01 vs. sham group. ^#^*p* < 0.05 and ^##^*p* < 0.01 vs. CLP group.

Further, the expression of molecules related to the NF-*κ*B signaling pathway was detected using western blot and RT-qPCR, respectively. The results of western blot (Figures 6(a) and 6(b)) and RT-qPCR (Figure 6(c)) showed that compared with the sham group, the expression of TLR4, NF-*κ*B, and Bax in the small intestine in the CLP group was significantly increased, while the expression of occludin and Bcl-2 was significantly decreased. However, a decrease in TLR4, NF-*κ*B, and Bax expression and an increase in occludin and Bcl-2 expression were identified after ulinastatin and lentinan treatment. And this change caused by lentinan was also dose-dependent. These results suggested that lentinan could effectively inhibit the activation of intestinal NF-*κ*B signal in the CLP mice and inhibit the apoptosis of small intestine epithelial cells.

## 4. Discussion

Sepsis, as a severe systemic inflammatory response syndrome, is one of the main causes of multiple organ dysfunctions [20]. Gut-origin sepsis leads to suppression of intestinal immune function and destruction of intestinal mucosa integrity, thus resulting in impaired intestinal barrier function, intestinal bacterial translocation, and an excessive inflammatory reaction in the host [21]. Therefore, it is essential to study the mechanism of inflammatory response and oxidative stress in gut-origin sepsis.

Most patients with sepsis are characterized by chronic inflammation and secondary infection [5]. In the presence of sepsis in the intestine, endotoxin stimulates intestinal mucosal mast cells to release TNF-*α*, which in turn triggers an inflammatory cascade by activating macrophages and produces a large number of proinflammatory cytokines to promote inflammation [4]. Proinflammatory cytokines, including IL-1*β*, IL-6, and HMGB1, mediate the inflammatory response of immune cells and promote systemic inflammatory response syndrome [22]. The results of this study showed that gut-origin sepsis was associated with increased expression of the proinflammatory cytokines (TNF-*α*, IL-1*β*, IL-6, and HMGB1) and decreased expression of anti-inflammatory cytokine IL-10. Moreover, in mice with gut-origin sepsis, their lung, liver, and ileal tissues showed obvious inflammatory injury, and serum liver function indicators (ALT, AST, and TBIL) were significantly increased. These results indicated that gut-origin sepsis could lead to a systemic inflammatory response and cause inflammatory injury to multiple organs. Lentinan has been reported to be an immunomodulator and to improve inflammatory injury of the intestine. It has antioxidant capacity in animals and plays an important role in maintaining intestinal barrier homeostasis [13, 16]. In this study, lentinan effectively improved the inflammatory injury of multiple tissues in mice with gut-origin sepsis, reduced proinflammatory cytokines, and alleviated the inflammatory response of the body.

In addition, the aggravation of sepsis also triggers an imbalance of reactive oxygen species (ROS) levels in organs [23] and therefore promotes cytokine release and oxidative stress injury in organs [24]. The results of this study showed a significant increase of the oxidative damage indexes (MPO, MDA, and SOD) in mice with gut-origin sepsis, while lentinan could reduce the oxidative damage. In severe sepsis, cytokines and chemokines are associated with the interruption of neutrophil migration at the site of infection [21, 25, 26] and may be involved in bacterial translocation during sepsis infection. Our study confirmed that lentinan could effectively reduce the liver injury indexes in mice with gut-origin sepsis and significantly reduce bacterial translocation in multiple tissues.

Gut-origin sepsis is often accompanied by severe intestinal inflammatory injury [21]. Studies have confirmed that the activation of NF-*κ*B triggers the release of proinflammatory cytokines, thereby further aggravating the inflammatory response and mediating cell apoptosis [27, 28]. By contrast, the inhibition of the NF-*κ*B signaling pathway improves intestinal injury in mice with sepsis [29]. Therefore, we speculated that lentinan might protect intestinal epithelial cells by inhibiting the NF-*κ*B signaling pathway. We confirmed experimentally that lentinan could significantly reduce the protein expression of NF-*κ*B as well as TLR4, NF-*κ*B, and Bax, but increase the expression of occludin and Bcl-2 in intestinal tissue of mice with gut-origin sepsis. These indicated that lentinan could reduce intestinal epithelial cell apoptosis by inhibiting the NF-*κ*B signaling pathway, which in turn improved intestinal injury. The protective effect of lentinan on mice with gut-origin sepsis had dose-dependent effect, and the efficacy of high concentration of lentinan was even better than that of ulinastatin in this study. However, there are some limitations in this study. Further experimental studies, therefore, are needed on the specific mechanism of how lentinan inhibits the NF-*κ*B signaling pathway to protect mice with gut-origin sepsis and the role of intestinal microbiota in this regard.

## 5. Conclusion

In summary, lentinan has a protective effect on mice with gut-origin sepsis, mainly by reducing the inflammatory response and oxidative stress to reduce injuries of the intestinal mucosa, lung, and liver. Lentinan can also reduce apoptosis of small intestinal epithelial cells by inhibiting the NF-*κ*B signaling pathway, thus improving intestinal injury.

## Figures and Tables

**Table 1:** Comparison of bacterial translocation rate in organs of mice in each group.

	*N*	Mesenteric lymph node	Lung	Liver	Bacterial translocation rate (%)
Sham	10	1	0	0	10.0
CLP	10	3	2	2	70.0^∗∗^
UTI	10	2	0	2	40.0^#^
LTN-L	10	3	1	2	60.0^#^
LTN-M	10	2	1	1	40.0^#^
LTN-H	10	2	0	1	30.0^##^

^∗∗^ *p* < 0.01 vs. sham group; ^#^*p* < 0.05 and ^##^*p* < 0.01 vs. CLP group. CLP: cecal ligation and perforation; UTI: ulinastatin; LTN-L: 5 mg/kg lentinan; LTN-M: 10 mg/kg; LTN-H: 20 mg/kg.

## Data Availability

The data used to support the findings of this study cannot be made freely available. Requests for access to these data should be made to Hang Xu, shihezizy@163.com.
